# Preparation, Characterization, and Environmental Safety Assessment of Dithiocarbazate Loaded Mesoporous Silica Nanoparticles

**DOI:** 10.3390/nano13020370

**Published:** 2023-01-16

**Authors:** Thacilla Menezes, Sirine Bouguerra, Tatiana Andreani, Ruth Pereira, Carlos Pereira

**Affiliations:** 1 Porto University Chemistry Research Center (CIQUP), Department of Chemistry and Biochemistry, Institute of Molecular Sciences (IMS)—Faculty of Sciences of the University of Porto (FCUP), Rua do Campo Alegre 687, 4169-007 Porto, Portugal; 2 GreenUPorto—Sustainable Agrifood Production Research Centre & INOV4AGRO, Faculty of Sciences of the University of Porto (FCUP), Rua do Campo Alegre s/n, 4169-007 Porto, Portugal; 3 Centre for Research and Technology of Agro-Environmental and Biological Sciences (CTAB) & INOV4AGRO, University of Trás-os-Montes e Alto Douro (UTAD), 5000-801 Vila Real, Portugal

**Keywords:** Schiff bases, loading, nanomaterials, ecotoxicology, non-target aquatic species, algae, plant, bacteria

## Abstract

Dithiocarbazates comprise an important class of Schiff bases with remarkable biological applications due to the imine group present in their structure. However, full exploitation of the biological activity of 3-methyl-5-phenyl-pyrazoline-1-(*S*-benzyldithiocarbazate) (DTC) is limited due to its easy degradation and poor solubility in aqueous solutions. The loading of DTC into mesoporous silica nanoparticles (MSiNPs) can be an excellent strategy to improve the solubility of DTC in the aqueous medium. Therefore, the main goal of the present work was to design MSiNP-DTC and to evaluate the success of the loading process by measuring its physicochemical properties and evaluating the environmental safety of the new DTC formulation using different aquatic organisms, such as the microalgae *Raphidocelis subcapitata*, the macrophyte *Lemna minor*, and the marine bacterium *Aliivibrio fischeri.* DTC, MSiNP, and MSiNP-DTC concentrations ranging from 8.8 to 150 mg L^−1^ were tested for all the species, showing low toxicity against aquatic organisms. Loading DTC into MSiNPs caused a slight increase in the toxicity at the concentrations tested, only allowing for the estimation of the effect concentration causing a 20% reduction in bioluminescence or growth rate (EC_20_). Therefore, despite the potential of MSiNPs as a drug delivery system (DDS), it is of utmost importance to assess its impact on the safety of the new formulations.

## 1. Introduction

Schiff bases (imine or azomethine) are an extensive group of organic compounds that have been widely used in the industrial sector since their first report by Hugo Schiff in 1864 [1]. These compounds are characterized by the typical presence of the bond >C=N (derived from the condensation of primary amines with aldehydes or ketones), with significant biological and pharmacological properties [2], including antifungal, antibacterial, antiproliferative, anti-inflammatory, antiviral, and antitumor [3,4]. Some in vitro studies have also shown that Schiff bases can be effective in treating neglected diseases, such as malaria, trypanosomiasis, and tuberculosis, compared to drugs already on the market, which cause more side effects due to the high concentrations needed to respond to resistances [5,6].

One notable example of a compound belonging to this class is 3-methyl-5-phenyl-pyrazoline-1-(*S*-benzyldithiocarbazate) (DTC), which exhibits a vast range of biological activities and has been shown to be interesting for the control of microbial agents and pathogens [3]. However, DTC presents low solubility and stability in aqueous media, limiting its administration and bioavailability. Such limitations can be overcome using drug delivery systems (DDS), which can protect the drug against premature degradation, improve drug solubility, and control its release, thus contributing to the highest efficiencies and decreasing application doses [7,8,9].

Mesoporous silica nanoparticles (MSiNPs) have been extensively investigated in the scientific community for producing DDS, serving as a host material to encapsulate or adsorb active ingredients or therapeutic agents [10,11,12,13], offering a pore network with homogeneous size (50 to 3000 nm), and providing cavities that can host and release a wide variety of molecules. MSiNPs have a uniform and adjustable particle size/shape and are biocompatible [14,15]. Numerous studies aiming at assessing the ecotoxicity of silica nanoparticles (SiNPs) have been published. However, frequently, the results have been controversial and contradictory, which may result, among other aspects, in authors testing particles with a common designation but with different properties, as it was shown by Andreani et al. [16]. The toxicity of SiNPs to aquatic organisms depends on several factors, especially on the physicochemical properties of the NPs, such as the morphology, average particle size, surface area, and surface charge, which in turn may affect the dispersibility, aggregation, and sedimentation of the tested NPs in an aqueous culture media of the test species [17]. Some studies have shown that the toxicity of SiNPs is size-dependent, with smaller particles (smaller than 100 nm) showing greater toxicity [18]. For example, Book et al. [19] recorded that SiNPs were not toxic to the bacterium *Pseudomonas putida* and the microalgae *R. subcapitata* (concentration range of 5–500 mg L^−1^) and to the crustacean *Daphnia magna* (>1000 mg L^−1^), except for SiNPs with an average size of 17 nm, which caused a slightly toxic effect on the algae (EC_20_ = 295 mg L^−1^). SiNPs with sizes of 50 nm and 195.5 nm up to a maximum concentration of 20 mg L^−1^ were also not toxic to *L. minor*, according to Andreani et al. [16]. A study with adult zebrafish females showed no physiological or morphological changes after the administration of SiNPs at a concentration of 25 mg L^−1^ and a relatively long exposure (30 days). However, there were significant changes in females’ ovarian structure and a significant inhibition of the eggs’ maturation, reflecting the impact of SiNP accumulation in the body, with subsequent effects on reproduction [20].

Although there are no data on the effect of dithiocarbazates or DTC on aquatic organisms, some ecotoxicity studies have been carried out with dithiocarbamates that are also Schiff bases, which are widely used as pesticides. The effect of the pesticide Mancozeb (MZ), a manganese/zinc ethylene bis-dithiocarbamate, was evaluated against *D. magna.* The toxicity of MZ to aquatic organisms was reported to be moderate to high with a 48 h EC_50_ value for 1000 µg L^−1^ of *D. magna* [21]. Zebrafish embryos (*Danio rerio*) were also exposed to Mancozeb up to a maximum concentration of 20 µg L^−1^. The results showed that there were significant changes in the mobility and morphology, as well as in the growth of *D. rerio* after the embryos’ exposure to MZ at all exposure times: 24, 28, 72, and 168 h post-fertilization (hpf). Changes in the levels of reactive oxygen species (ROS) and antioxidant enzyme activity, such as glutatione S-transferase (GST), catalase (CAT), and superoxide dismutase (SOD), were observed only at 72 and 168 hpf. Exposure to MZ promoted an increase (*p* < 0.001) in the ROS steady-state levels at 168 hpf in 20 µg L^−1^, and also caused a significant inhibition (*p* < 0.0001) of the GST activity in all concentrations tested compared to the control group. The CAT activity was increased (*p* = 0.0151) and the SOD activity remained unchanged [22].

Fred et al. [23] also studied the effect of exposure to some dithiocarbamate compounds (pyrolidine-DTC (PDTC), dimethyl-DTC (DMDTC), sodium metam (NaM), methylisothiocyanate (MITC)), and their main degradation product, carbon disulfide (CS_2_), to zebrafish. In this study, all tested dithiocarbamates caused notochord distortions with notochord NOELs of <4 to 40 ppb for a maximum exposure time of 24 hpf, while CS_2_ also caused distortions at concentrations 200 times greater than that tested for dithiocarbamates. Another pesticide from the dithiocarbamate group, Thiram, widely used to prevent fungal diseases in seeds and crops, was shown to decrease zebrafish embryo survival at concentrations up to 10 μM after 24 h of exposure [24].

Therefore, the present work aimed at: (i) developing a hybrid nanostructure based on MSiNPs loaded with DTC; (ii) characterizing the morphology and the physicochemical properties of MSiNP-DTC in several aqueous media and the DTC loading efficacy, and (iii) assessing the toxicity of MSiNPs non-loaded and loaded with DTC (MSiNP-DTC) on different aquatic species, such as *A. fischeri*, *R. subcapitata* and *L. minor*, using standard protocols. To the best of our knowledge, there has been no report regarding the effect of DTC or MSiNP-DTC on aquatic organisms. MSiNPs present several advantages as DDS for the loading of DTC for antimicrobial purposes, with possible applications in medicine, the textile industry, or even agriculture. MSiNPs loaded with DTC may be released to the environment either as a consequence of its fabrication process or its applications, affecting biological communities, and therefore, it is of utmost importance to assess its safety for a battery of aquatic species.

## 2. Materials and Methods

### 2.1. Reagents

Hexadecyltrimethylammonium bromide (CTAB, ≥99%), tetraethyl orthosilicate (TEOS, ≥99%), absolute ethyl alcohol (Fermont, ≥99.5%), N_2_H_4_.H_2_O (≥98%), CS_2_ (anhydrous, ≥99%), PhCH_2_Cl (≥99%), HCl (≥37%), MeOH (anhydrous, ≥99.8%), and 4-phenyl-2,4-butanedione were supplied by Sigma-Aldrich. Ultrapure water was obtained from the Milli Q Plus system (Millipore^®^, Darmstadt, Germany).

### 2.2. Synthesis of DTC

**Figure 1:**
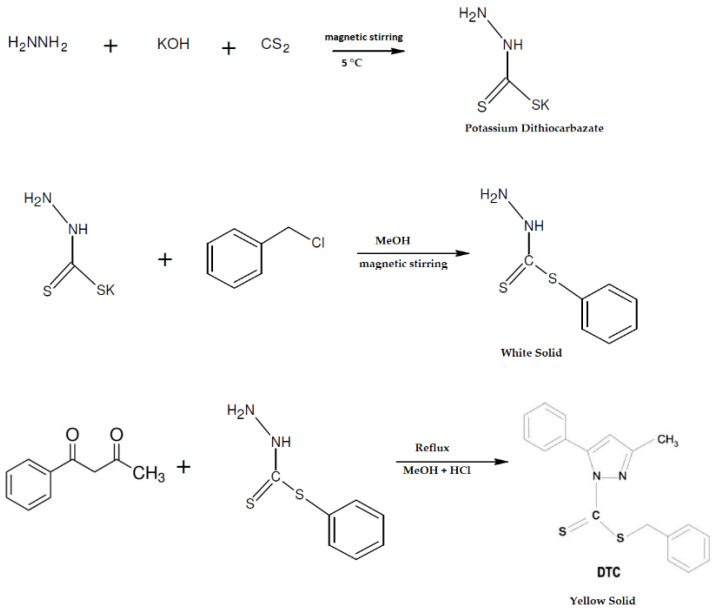
Schematic representation of the synthesis process of 3-methyl-5-phenyl-pyrazoline-1-(*S*-benzyldithiocarbazate) (DTC).

DTC (C_18_H_16_N_2_S_2_) was synthesized according to previously reported procedures (Figure 1) [25]. KOH (11.4 g) was dissolved in 90% ethanol, N_2_H_4_.H_2_O (10.2 mL) was added, and the mixture was cooled to 0 °C in an ice-salt bath. CS_2_ (19.24 mL) was added dropwise under constant and vigorous stirring (700 r.p.m) for 1 h. Subsequently, two layers were observed, one upper yellow and a lower brown layer; this lower layer was separated and dissolved in 40% cold ethanol, keeping the reaction between 5 and 7 °C. Once the temperature was maintained, PhCH_2_Cl (27.5 mL) was added dropwise with vigorous stirring for 30 min. The white solids that were formed were filtered, washed with ultrapure water, and, after drying at room temperature, it was recrystallized in methanol and dried over anhydrous CaCl_2_ (total mass obtained = 6.2238 g). Next, a solution of 1-phenyl-1,3-butanedione (0.811 g) in methanol (10 mL) was added to a solution of S-benzyldithiocarbazate (0.991 g) in methanol (10 mL) under reflux with the addition of two drops of HCl 37% to the reaction medium, employing an acid-catalyzed conversion and maintaining reflux for 2 h. A yellow solid compound was obtained, subsequently filtered, washed with methanol, and dried. The product was also recrystallized in methanol to obtain pure yellow crystals (as reported by De Sousa et al. [25]). The total mass obtained = 0.8728 g and yield = 95.81%.

### 2.3. Synthesis of MSiNPs and MSiNP-DTC

**Figure 2:**
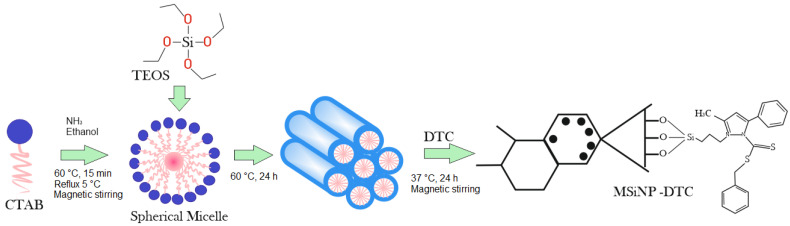
Schematic representation of the synthesis process of MSiNPs and MSiNP-DTC (Adobe Photoshop CC2014).

The synthesis of MSiNPs was carried out under a high concentration of precursors, resulting in NPs with a highly uniform spherical shape (Figure 2). For this procedure, 750 mg of cetyltrimethylammonium bromide (CTAB) was dissolved in 20 mL of an aqueous solution of NH_3_ (0.048 mol L^−1^), followed by homogenization under magnetic stirring (300 r.p.m) in a round-bottom distillation flask connected to a condenser reflux setup at 5 °C (to prevent the evaporation of the ethanol). To this solution, 3.2 mL of absolute ethanol was added as a co-solvent, and the mixture was homogenized for 15 min at 60 °C. Sequentially, 2.5 mL of tetraethyl orthosilicate (TEOS) (1.2 mmol) was added to the solution and the flask was kept at the same temperature for 2 h with agitation (300 r.p.m). At the end of the reaction, the MSiNPs were separated from the solution by centrifugation and washed with absolute ethanol before the extraction of the soft mold (CTAB). Finally, the MSiNPs were washed twice with absolute ethanol and dried at 60 °C for 24 h. The residual CTAB was removed in an inert atmosphere (N_2_) at 550 °C, with a temperature increase rate of 3 °C min^−1^ for 5 h [26]. For the MSiNP-DTC, 5 mg of DTC was solubilized in 10 mL of ethanol at 37 °C, followed by the addition of 100 mg of MSiNPs under magnetic stirring (300 r.p.m) for 2 h. Then the suspension was centrifuged at 15.000 r.p.m. The MSiNP-DTC was washed with water, separated by a second centrifugation, and dried at 60 °C overnight, making it available for analysis as a powder.

## 3. Physicochemical Characterization of MSiNPs and MSiNP-DTC

### 3.1. Average Particle Size, Polydispersity Index, and Zeta Potential Analysis

The average particle size (Z-Ave) and polydispersity index (PDI) of the prepared MSiNPs and MSiNP-DTC were determined by dynamic light scattering (DLS) using a Malvern Zetasizer Nano ZS90 (Malvern, UK), at 25 °C. The zeta potential (ZP) was determined by applying an electric field to the samples, and the ZP values were obtained by measuring the velocity of the electrophoretic mobility of the particles using the Doppler laser anemometry technique (Malvern Zetasizer Nano ZS (Malvern Instruments, Works, UK), at 25 °C. The MSiNPs and MSiNP-DTC were suspended in ultrapure water (200 mg L^−1^) by sonication for 10 min in an ultrasonic bath (Ultrasonic Cleaner, SONICA^®^, Soltec, Milan, Italy). Afterwards, 1 mL of the suspension was used for the analysis. The Z-Ave, PDI, and ZPs of the dispersions in Woods Hole MBL (*R. subcapitata* culture medium) and Steinberg medium (of *L. minor*) were also determined. The data for each parameter were expressed as the mean ± standard deviation (SD) of three measurements.

### 3.2. Transmission Electron Microscope (TEM) Analysis

The morphology of the NPs was checked by transmission electron microscopy (TEM) (Hitachi model H8100, with an LaB6 filament and using an accelerating voltage of 200 kV). Images were acquired with an Olympus Keenview CCD camera (formerly Soft Imaging). The MSiNPs and MSiNP-DTC were dispersed in ultrapure water (conductivity 0.14 µS cm^−1^) by sonication for 30 min in an ultrasonic bath and placed on copper grids covered with a carbon coating for TEM observations.

### 3.3. Fourier Transform Infrared (FT-IR)

The FT-IR spectra were obtained using a Bruker Vector 22 spectrometer in the 400–4000 cm^−1^ region. Loaded and unloaded MSiNPs were mixed with a suitable amount of micronized KBr powder and compressed into disks with a force of 10 kN using a manual press to prepare the tablet. For each spectrum, 100 scans were collected with a resolution of 4 cm^−1^ in the medium infrared region at 25 °C.

### 3.4. Thermal Analysis

Thermal characterization was used to identify the thermal decomposition of the MSiNPs and MSiNP-DTC, performed by differential scanning calorimetry (DSC) and simultaneous thermal analysis (thermogravimetry (TG) and differential thermal analysis (DTA)) techniques. The DSC studies were performed using a DSC 7020 (Hitachi High Tech Science Corporation, Tokyo, Japan). The analysis was performed with about 10 mg of MSiNPs and MSiNP-DTC placed in an aluminum pan (7.5 µL), which was hermetically sealed. Scans were carried out from 25 to 600 °C at a heating rate of 5 °C min^−1^ under nitrogen atmosphere (100 mL min^−1^). The TG and DTA analyses were performed using simultaneous thermal analysis (STA) (STA 7200RV, Hitachi High-Tech Science Corporation, Tokyo, Japan). The NPs were accurately weighed in aluminum pans, and the scans were performed from 25 to 400 °C at a heating rate of 10 °C min^−1^ under nitrogen atmosphere (200 mL min^−1^).

### 3.5. Loading Efficiency

Briefly, 5 mg of MSiNP-DTC was dispersed in 1 mL of ethanol and centrifuged at 13.500 r.p.m for 30 min. Then, 200 µL of supernatant was removed and diluted with ethanol to 500 µL and analyzed by UV-visible spectroscopy at 303 nm using a T70 UV-VIS Spectrometer (PG Instruments Ltd., Leicestershire, UK) for the quantification of DTC residues. The data were expressed as the mean ± SD of three measurements. The entrapment efficiency (% EE) and the drug load (% DL) were determined by Equations (1) and (2), respectively.

(1) $$\%\;\mathrm{EE}=\frac{\left(\mathrm{Amount}\;\mathrm{of}\;\mathrm{initial}\;\mathrm{DTC}\;-\;\mathrm{Amount}\;\mathrm{of}\;\mathrm{free}\;\mathrm{DTC}\right)}{\mathrm{Amount}\;\mathrm{of}\;\mathrm{initial}\;\mathrm{DTC}}\times 100$$

(2) $$\%\;\mathrm{DL}=\frac{\left(\mathrm{Amount}\;\mathrm{of}\;\mathrm{initial}\;\mathrm{DTC}\;-\;\mathrm{Amount}\;\mathrm{of}\;\mathrm{free}\;\mathrm{DTC}\right)\;}{\;\mathrm{Total}\;\mathrm{Nanoparticle}}\times 100$$

### 3.6. Nitrogen Adsorption

The surface areas of the different mesoporous silica grades were determined by nitrogen adsorption at −196 °C using a TriStar II Plus 2.02 from Micrometrics. The specific surface areas (SBETs) were calculated using the Brunauer–Emmett–Teller (BET) method [27]. The pore size distribution and pore volume were estimated from the desorption branch of the N_2_ adsorption–desorption isotherms by applying the Barrett–Joyner–Halenda (BJH) method [28]. The measurements were performed in triplicate for the MSiNPs and MSiNP-DTC in powder, and the data were presented as the mean ± SD.

## 4. Ecotoxicological Assays

### 4.1. Preparation of Solutions and Dispersions

As DTC is not soluble in aqueous media, 0.5% (*v*/*v*) DMSO in water was used as a solubilizing agent for the DTC. DMSO in an aqueous solution was previously tested on *A. fischeri* at 5.0% (*v*/*v*) [29] and on *R. subcapitata* at 1% (*v*/*v*), showing no toxicity for either species [30].

For the growth inhibition tests of *R. subcapitata* and *L. minor*, a stock solution/dispersion of MSiNPs and MSiNP-DTC was prepared at a concentration of 200 mg L^−1^ in ultrapure water, followed by an ultrasound bath for 10 min to disperse the nanoparticles. The amount of DTC within 200 mg L^−1^ of MSiNPs was 9.6 mg L^−1^. A DTC stock solution (200 mg L^−1^) was prepared by dissolving DTC in 0.5% (*v*/*v*) DMSO in water. Subsequently, the stock solutions/dispersions of DTC, MSiNPs, and MSiNP-DTC were diluted in Woods Hole MBL and Steinberg culture media directly on the plates according to the concentrations used in the assays: 0.0, 8.8, 13.2, 19.8, 29.6, 44.4, 66.7, 100, and 150 mg L^−1^. For the *Microtox* assays, the same procedure for preparing the stock solutions used for *R. subcapitata* and *L. minor* was used. For the MSiNPs and MSiNP-DTC, this concentration was based on the amount of MSiNPs. Thus in 150 mg L^−1^ of MSiNPs, there was 7.18 mg L^−1^ of DTC, considering the loading efficiency of DTC in the nanoparticle.

### 4.2. Microtox Test

The toxicity test with *A. fischeri* bacteria was performed using the Microtox 500 Analyzer (Modernwater, Newcastle, DE, USA) following the 81.9% basic test protocol of AZUR [31]. The bioluminescence of the bacteria was measured after 5, 15, and 30 min of exposure. The EC_50_ and EC_20_ values for bioluminescence inhibition were computed for each contact time using Azotox^®^ MicrotoxOmni software version V1.18.

### 4.3. Growth Inhibition Test with R. subcapitata

An *R. subcapitata* growth inhibition assay was performed according to the standard OECD protocol 201 [32]. The microalgae were obtained from axenic batch cultures maintained in the Woods Hole MBL medium at continuous light exposure (cool white fluorescent illumination of 100 µE·m^−2^ s^−1^) and a temperature of 21 ± 2 °C. The algae were exposed in 24-well sterile plates in three replicates (wells) per concentration, plus three replicates for the control. In each well, 900 µL of the dispersions was added at different concentrations, followed by the addition of 100 µL of algal inoculum to an initial cell concentration of 10^4^ cells mL^−1^. The microplates were maintained in continuous agitation for a period of 72 h under the same conditions described for the culture maintenance. The cells were resuspended manually with a micropipette twice a day. The cells of each replicate were counted using a Neubauer chamber in an optical microscope. The average specific growth rate for each treatment and chemical compound was calculated using the following equation:

$$\mu_{ij}=\frac{ln\left(X_{j}\right)-\ln(X_{i})}{t_{j}-t_{i}}\;\left({\mathrm{day}}^{-1}\right)$$

where

- *µ_ij_* is the average specific growth rate from time *i* to *j*;
- *X_i_* is the biomass at time *i*;
- *X_j_* is the biomass at time *j*;
- *t* is the time period from *i* to *j*.

### 4.4. Growth Inhibition Test with L. minor

An *L. minor* growth inhibition test was performed according to the OECD guideline 221 [33]. Plants were cultured in the laboratory in Steinberg medium with a pH adjusted to 7.5 ± 0.1. The cultures were maintained in axenic condition, with a light intensity of 6.000–10.000 Lux obtained from cool white fluorescent illumination and a temperature of 21 ± 2 °C. The test was run in a 16^L^:8^D^ h photoperiod at 20 ± 1 °C for 7 days. A total of four colonies with three visible fronds each were initially exposed per replicate (four replicates per concentration plus the control). The exposures were carried out in sterilized 6-well microplates, filled with 12 mL of each treatment/control. After 7 days of exposure in the same conditions described for the culture maintenance, all the fronds were collected from each microplate well, counted, and dried at 60 °C to a stable weight. The effects of the DTC, MSiNPs, and MSiNP-DTC were evaluated based on the growth rate of the *L. minor*, expressed as the number and dry weight of the fronds after 7 days of exposure. The average specific growth rate for each treatment and chemical compound was calculated, taking into account both the number of fronds and the dry weight and using the same equation described above.

### 4.5. Statistical Analysis

For all experiments, the data were reported as the mean ± standard deviation (SD). Statistically significant differences among the treatments for each assay and parameter assessed were tested by univariate analysis of variance (ANOVA), with a subsequent Dunnett’s post-hoc test to determine the differences among all treatments (DTC, MSiNPs, and MSiNP-DTC concentrations of 0.0, 8.8, 13.2, 19.8, 29.6, 44.4, 66.7, 100, and 150 mg L^−1^)) and the control group. No observed effect concentrations (NOECs) and low observed effect concentrations (LOECs) were determined based on the previously described analysis. The EC_50_ and EC_20_ values were determined by applying non-linear least square regression analysis using STATISTICA^®^ 7.0 software (StatSoft, Inc., Tulsa, OK, USA). A level of significance α = 0.05 was used. Prior to these statistical analyses, the assumptions of normality and homoscedasticity were checked using Kolmogorov–Smirnov and Levene’s tests (for *p* < 0.05), respectively. All graphs were created using Prisma GraphPad 8 software (La Jolla, CA, USA).

## 5. Results and Discussion

The results of the characterization of both the MSiNPs and MSiNP-DTC suspended in water and in different assays’ culture media are described in Table 1. It is shown that the Z-Ave ranged from 168 ± 4 nm for unloaded MSiNPs to 176 ± 1 nm for MSiNP-DTC. Concerning the PDI, the obtained values for both the unloaded and loaded nanoparticles point to monodispersed suspensions, with average values of 0.29 ± 0.02 and 0.38 ± 0.04, respectively. The ZP values for the MSiNPs changed from positive 16.8 ± 0.2 mV to negative values −11.7 ± 0.4 mV after calcination in a nitrogen atmosphere due to the neutralization of the ionizable fraction (residual silanol groups (Si-OH)) of the MSiNPs. After loading with DTC, higher |ZP| values (−21.9 ± 0.3 mV) were observed in comparison to the unloaded MSiNPs, thus suggesting that the loaded MSiNP-DTC had the highest stability.

The data of the same physico-chemical and morphological parameters in suspensions obtained in different culture media showed a slight increase in the Z-Ave and the polydispersity index. The most remarkable increase was recorded for the MSiNP-DTC in the Steinberg medium. It is generally accepted that smaller particles can easily penetrate cell membranes and thus induce cytotoxicity. Therefore, the relationship between size and toxicity is still controversial, as one study reported that size played no role in toxicity, as smaller NPs were less toxic than their counterparts with a higher Z-Ave [34]. The aggregation of NPs depends on their particle concentration, pH, ionic strength, ionic composition and concentration, and other characteristics of the aqueous media [35]. The observed aggregation of MSiNPs in Steinberg medium compared to water can be attributed to the possible higher ionic strength of this medium, which reduced the stability of the NPs in suspension (low |ZP|). In the case of the MSiNP-DTC, the modification of the silica surface with DTC led to the formation of large agglomerates in alkaline solution in Steinberg medium (pH = 7.5 ± 0.1), which were almost twice as large as in neutral and acid solutions. This may be related to the dissociation of hydrogen bonds on the surface of MSiNPs and the delocalization of π (pi) electron pairs from the C-N and C-S bonds of the DTC, leading to a strong interaction between the MSiNPs and DTC and resulting in an increase in the ionic strength [36,37].

**Figure 3:**
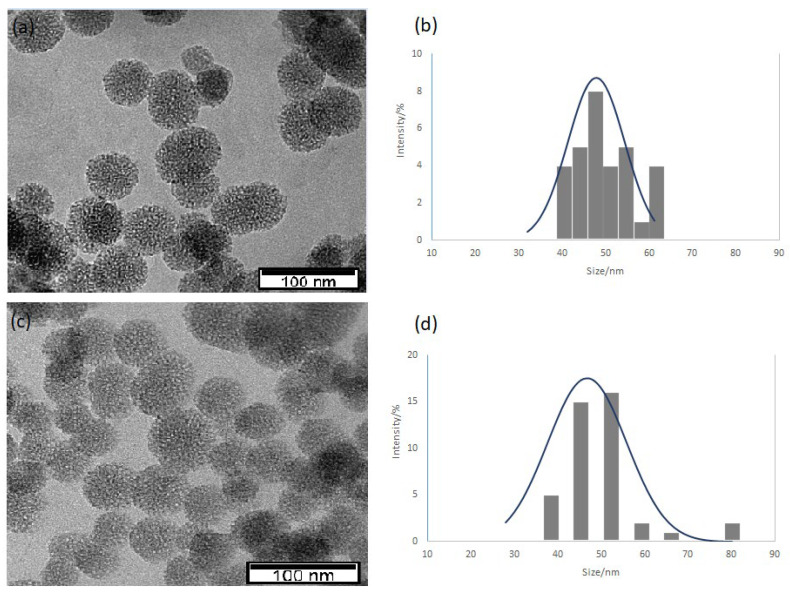
TEM images of (**a**) MSiNPs and (**c**) MSiNP-DTC NPs and their respective size distribution histograms (**b**,**d**).

TEM was used to evaluate the size distributions, structure, and morphology of the MSiNPs and MSiNP-DTC. Figure 3 shows the spherical form of the MSiNPs, with particle sizes of around 48 ± 5 nm for the MSiNPs and 47 ± 6 nm for the MSiNP-DTC. Furthermore, the TEM image clearly shows the mesoporous structure and that the incorporation of DTC had no impact on the size, shape, or morphology of the MSiNPs. The smaller Z-Ave observed in the TEM for both NPs does not contradict the Z-Ave values obtained by the DLS measurements since the size of dried structures analyzed by TEM is generally smaller than the same structure with a solvation layer around its surface, as analyzed by DLS [38].

**Figure 4:**
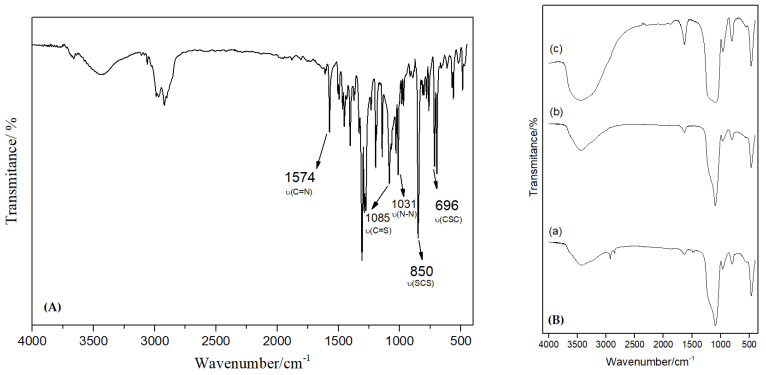
(**A**) Vibrational spectrum in the infrared region of the compound DTC. (**B**) Vibrational Spectrum in the infrared region of MSiNPs (a), MSiNPs calcined (b), and MSiNP-DTC (c) in KBr.

The FT-IR spectra of DTC, MSiNPs, and MSiNP-DTC are shown in Figure 4A,B, respectively. The DTC displayed two intense bands at 1574 and 1031 cm^−1^, which can be assigned to the ν(C=N) and ν(N-N) modes, respectively (Figure 4A). These bands are extremely important as they refer to the imine group, and a shift in the frequencies of these bands may be indicative of DTC loading in the silica matrix, in which a shift to lower frequencies is associated with a reduced binding order, while the shift to higher frequencies corresponds to an increase in the connection order. Figure 4B shows the spectra of MSiNPs (a), MSiNPs calcinated (b), and MSiNP-DTC (c). The absorption band around 1096 cm^−1^ was due to the vibrations of the asymmetric stretching of Si-O-Si, indicating the presence of silica in the MSiNPs, while the peaks at 807 and 468 cm^−1^ attributed to the groups Si-O-Si are symmetrical stretching and flexing vibrations, respectively. In the MSiNP-DTC spectrum, the appearance of new bands was not observed, but the main DTC bands were shifted, increasing the intensity of the bands related to the MSiNPs (Figure 4B(c)). Basically, at 1631 cm^−1^ the increase in the MSiNP peak intensity was due to the addition of the DTC band previously found at 1574 cm^−1^ of the ν(C=N) bond. The same happened with the broadening of the peak at 1099 cm^−1^ with the addition of the ν(C=S) and ν(N-N) bands from the DTC. Finally, at 796 cm^−1^ and 475 cm^−1^, the increase in intensity was due to the overlap of the ν(SCS) and ν(CSC) bands of the DTC to the peaks of the MSiNPs [39,40,41,42].

**Figure 5:**
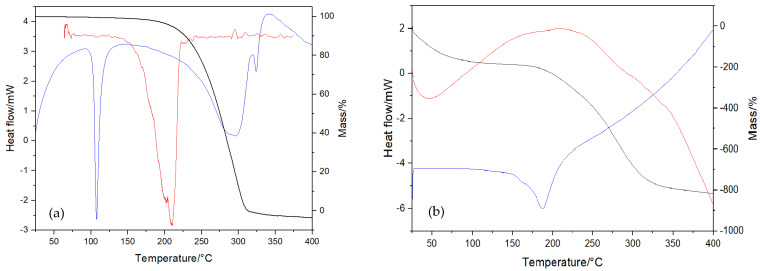
Thermal analysis of DTC (**a**) and MSiNP-DTC (**b**): thermogravimetry (TG) (%) (black line), differential thermal analysis (DTA) (red line), and differential scanning calorimetry (DSC) (blue line).

Thermal studies were performed within a temperature range of 25–600 °C under the dynamic atmosphere of nitrogen to investigate the thermal stability of the DTC and the synthesized nanoparticles (NPs). A previous study on DSC (Figure 5a) showed that DTC presented an intense and sharp endothermic peak at 107 °C, attributed to the melting process, and at 300 °C, associated with the process of decomposition or evaporation [5]. As the MSiNPs did not show any transition in the temperature range of 25–600 °C, only the thermal transition of MSiNP-DTC was observed (Figure 5b). Thus, a melting endothermic peak at around 104 °C was detected in the thermogram of MSiNP-DTC, confirming that DTC was incorporated into MSiNPs [43]. Similarly, Costa et al. [5] also showed a long decomposition step for DTC in the temperature range of 160–310 °C with a mass loss of around 95%. The NPs were also evaluated regarding their thermal stability using thermogravimetry (TG) from 20 to 400 °C, as represented for MSiNP-DTC (Figure 5b). Concerning the data obtained, the TG analysis showed that the mass loss for MSiNP-DTC was 12.52%. As shown in the thermal curves, the DTA thermograms of MSiNP-DTC show an intensive endothermic reaction in the range of 20–100 °C. This peak was correlated to the loss of physically adsorbed water from the surface and chemically adsorbed water bonded to Si-OH through the hydrogen bond.

Inorganic carriers, such as MSiNPs, tend to have a high loading capacity but low loading efficiency, unlike organic carriers, such as micelles, liposomes, and polymeric nanoparticles [44]. However, in this study, the MSiNP-DTC maintained an efficient drug load (4.79 ± 0.04%) and a high loading capacity (95.77 ± 0.08%), which may have resulted from strong electrostatic and hydrogen bonding interactions between the DTC and the silica matrix [45].

**Figure 6:**
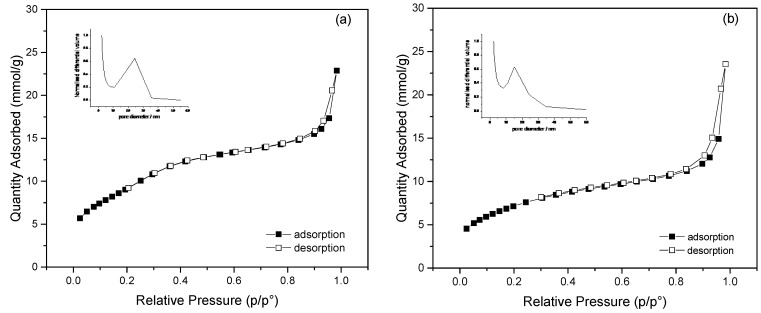
Nitrogen isotherms from MSiNPs (**a**) and MSiNP-DTC (**b**) and plots of the normalized differential volume as a function of pore diameter, as inset.

Figure 6a,b shows the adsorption–desorption isotherms of the MSiNPs and MSiNP-DTC, respectively, which correspond to type IV adsorption–desorption isotherms with an H1 hysteresis loop according to the IUPAC, typical for materials containing mesoporous structures with cylindrical pores [46,47]. The MSiNPs exhibited a high BET surface area of 1021 ± 15 m^2^ g^−1^, a pore volume of 1.61 ± 0.04 cm^3^ g^−1^, and a mean pore size of 24.3 ± 0.2 nm—all characteristics favorable for loading. For the MSiNP-DTC, the measured surface area and pore volume were estimated to be 618 ± 15 m^2^ g^−1^ and 1.01 ± 0.01 cm^3^ g^−1^, respectively, while the mean pore size calculated from the N_2_ adsorption–desorption isotherm was 15.2 ± 0.2 nm. Although such a change in the textural properties occurred after the DTC incorporation, the MSiNP-DTC still maintained the typical mesoporous structure during the immobilization process. The reductions in the surface area, volume, and mean pore size suggest that the DTC was present in the MSiNP matrix in various ways, such as adsorbed/adhered to the surface and inside the pore [41,48,49].

It is essential to understand how the different physicochemical characteristics of NPs may influence their toxicity, stability, and transformation in different environmental matrices. The Microtox^®^ assay is a sensitive test that is widely used to evaluate the toxic effects of chemical compounds or environmental samples [50]. The underlying principle of the *A. fischeri* bioluminescent assay is the correlation of changes in the kinetic attributes of the bioluminescent reaction after a brief contact of the bacteria with the NPs with their toxicity [51]. The decrease in the bioluminescence reflects the inhibition of bacterial metabolism and is proportional to the toxicity of the test sample [52]. *A. fischeri* is a Gram-negative bacterium, and the test is considerably fast, reproducible, and sensitive to a wide variety of toxic compounds [53].

Table 2 shows the % of the highest effect and EC_x_ values for *A. fischeri* after 5, 15, and 30 min of exposure. The concentration of the suspensions tested for the DTC, MSiNPs, and MSiNP-DTC was 150 mg L^−1^, which was then tested for different dilutions separated by a factor of two, according to the protocol of the basic test [31].

DTC is poorly soluble in aqueous media. In addition, it can undergo degradation, releasing by-products such as CS_2_, which was also reported as hazardous [23]. However, the DTC dissolved in 0.5% (*v*/*v*) DMSO in water was not toxic to *A. fischeri* (Table 1). This result indicates that there was no degradation of the DTC in the suspension, not even in the NaCl 2% medium used to dilute the bacteria. DTC in PBC buffer (300 mmol L^−1^) was not cytotoxic to mammalian cells by the blue thiazolyl tetrazolium bromide (MTT) assay, and it induced the production of nitric oxide (NO), a short-lived gas radical that is considered a molecule with important functions, such as regulating vascular tone, neurotransmission, acute and chronic inflammation, host defense mechanisms, cell viability modulator, and oxidative stress agents [3].

The toxicity of the MSiNP-DTC increased when compared to the non-loaded MSiNPs. However, the effect recorded was slightly above the 20% of bioluminescence inhibition after 30 min of exposure. According to the DLS data (Table 1), MSiNPs and MSiNP-DTC showed similar Z-Ave values (168 ± 4 and 176 ± 1 nm, respectively) and negatively charged particles. Thus, only the synergic interaction between the DTC and the MSiNPs could have accounted for a higher interaction of the NPs with the biological membranes, at least for the bacteria *A. fischeri*.

**Figure 7:**
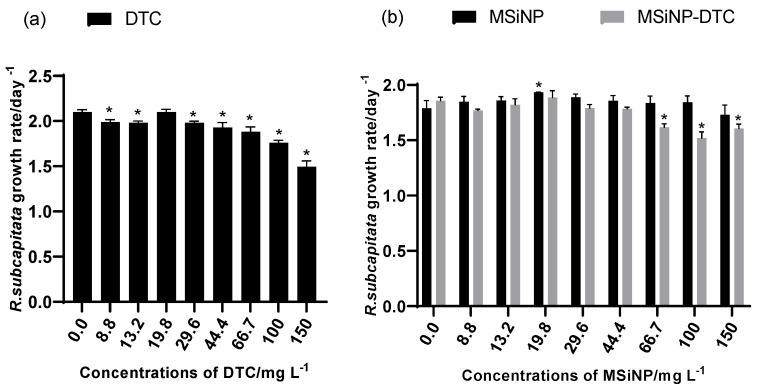
Average *R. subcapitata* growth rate after 72 h of exposure to DTC (**a**), MSiNPs, and MSiNP-DTC (**b**). Error bars represent the standard deviation, and * corresponds to significant differences between the respective control and the treatments (Dunnett’s test: *p* < 0.05). DTC: 3-methyl-5-phenyl-pyrazoline-1-(*S*-benzyldithiocarbazate); MSiNP: mesoporous silica nanoparticles; MSiNP-DTC: DTC-loaded silica nanoparticle. The concentrations of MSiNP-DTC are based on MSiNPs. The amount of DTC in 150 mg L^−1^ of MSiNPs is 7.18 mg L^−1^.

Microalgae are important model species to assess the toxicity of NPs, as they play a role in the aquatic ecosystem as primary producers that are vulnerable to alterations by contaminants [34]. Toxicity in algae is influenced by the inherent properties of NPs, such as their size, shape, concentration, and their coating layers, and these properties can lead to defensive behaviors of algae through the stimulation of the antioxidant system to remove reactive oxygen species (ROS) induced by NPs [54]. Previous authors proposed a classification of the toxicity of substances to microalgae based on the EC_50_ values recorded. This classification is called the Passino and Smith Classification (PSC), according to which the test substances are very toxic (EC_50_ < 1 mg L^–1^), toxic (1–10 mg L^–1^), harmful (10–100 mg L^–1^), or not harmful (>100 mg L^–1^) [55]. According to this classification, the MSiNPs proved to be non-toxic to *R. subcapitata* (Figure 7b), with only a 3.2% inhibition of the algae growth rate at the highest concentration tested. A study showed that the hydrophilicity/number of silanol groups on the particle surface was associated with SiNP toxicity, as proton-donating silanol groups denatured cell membrane proteins, leading to membrane damage [19]. However, at least for the concentrations tested in this study, such an effect was not recorded. As illustrated in Figure 7a, for the DTC (EC_20_ = 119.9 mg L^−1^), significant growth inhibition was recorded for almost all the concentrations tested up to a maximum of 29.6% of inhibition in the algae growth rate at the highest concentration (150 mg L^−1^) when compared to the control. Once it was an organic compound with an intense yellow color, the high concentration of DTC in the suspension probably indirectly caused the unavailability of light crucial for the growth of algae. The MSiNP-DTC was slightly toxic at the concentrations tested. The MSiNPs prevented the degradation of the DTC, and it was large enough to not cross the algal cell membrane or to agglomerate/aggregate in the aqueous medium, reducing the exposure of the *R. subcapitata* [56].

Several short-term toxicity assessments were performed on aquatic plant species. Duckweeds are a group of plants that have been used to determine the toxicity of NPs under laboratory conditions as a metal bioaccumulator and bioindicator for pollution detection and monitoring [57]. Duckweeds are easy to grow when there are adequate conditions of light, pH, temperature, and nutrient supply [58,59]. In particular, species of the genus *Lemna* are aquatic plant test species that are considered advantageous for being low-cost and less wasteful because the test procedure can be adapted to microplates [60]. In addition, the negative impact on duckweed can have serious consequences in the food chain, leading to changes in the diversity and also reflecting changes in the functionality of entire aquatic ecosystems [61]. Therefore, the ecotoxicity test with *L. minor* is now a methodology of choice for assessing the impacts of contaminants on freshwater systems [62].

**Figure 8:**
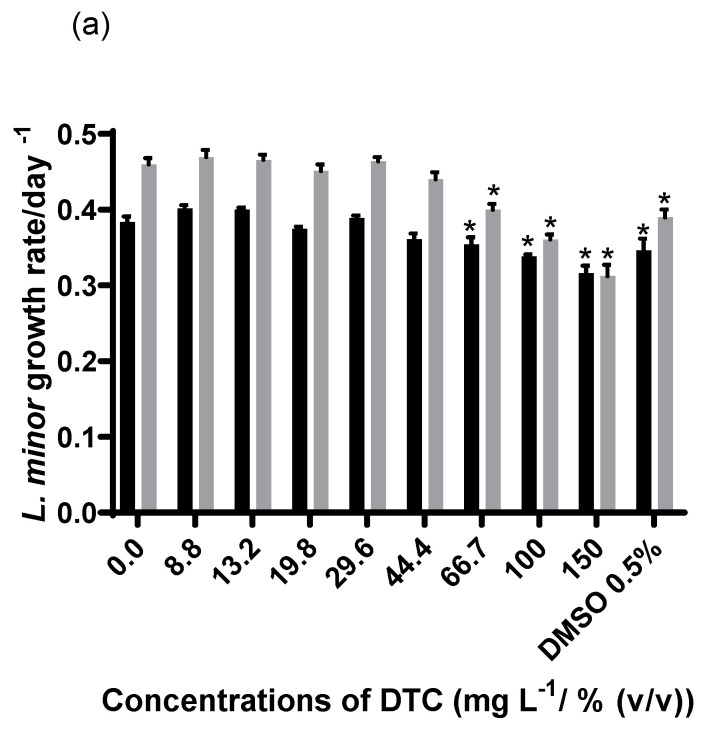

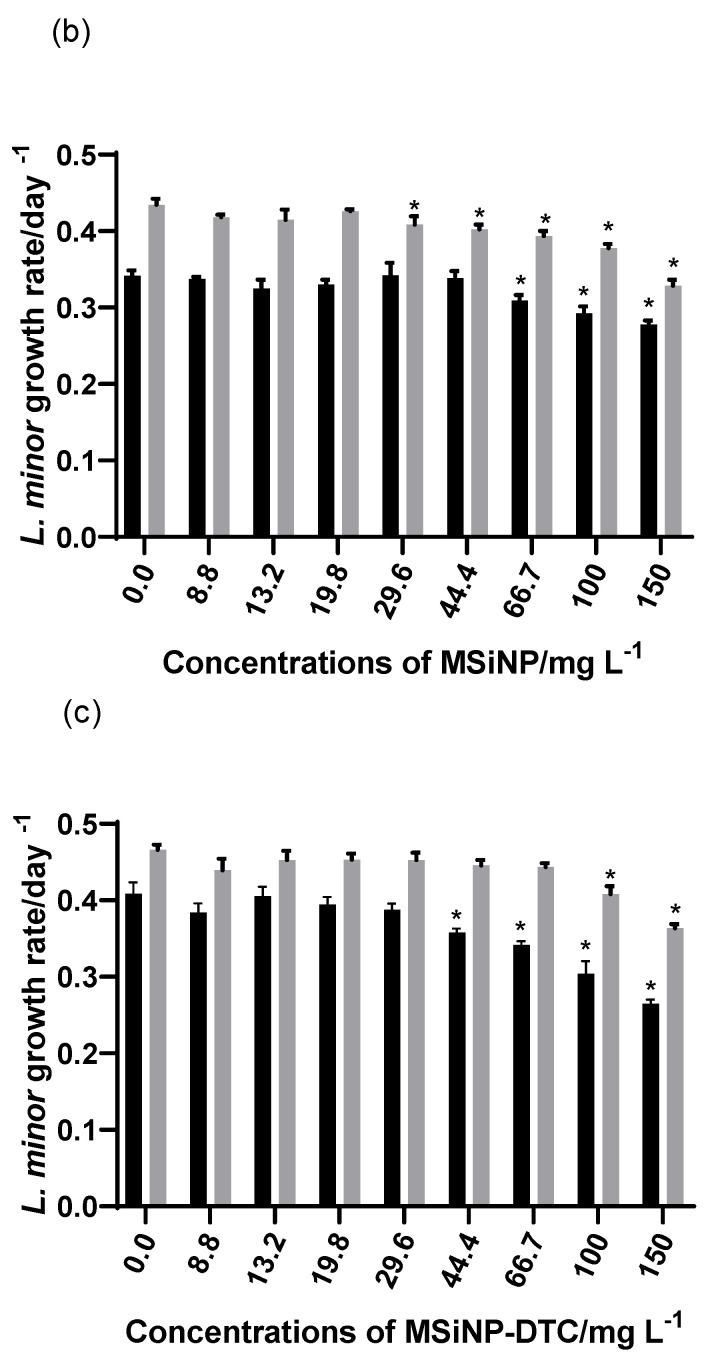
Average growth rate (day^−1^) calculated based on frond number (black bars) and on frond dry weight (grey bars) of *L. minor* exposed to DTC and DMSO 0.5% (*v*/*v*) (**a**), MSiNPs (**b**), and MSiNP-DTC (**c**). Error bars represent the standard deviation, and * corresponds to significant differences from the control (Dunnett’s test: *p* < 0.05). The concentrations of MSiNP-DTC are based on MSiNPs. The amount of DTC in 150 mg L^−1^ of MSiNPs is 7.18 mg L^−1^.

Currently, there are only a limited amount of data on the effect concentrations observed for *L. minor* regarding SiNPs [16,61], and none are related to DTC or analogous compounds. The effects of DTC, MSiNPs, and MSiNP-DTC on *L. minor* growth assessed by the number of fronds and dry weight after 7 days of exposure are shown in Figure 8 and Table 3. We can see that at the highest concentrations for both the DTC and SiNPs, there were significant effects on *L. minor*. For the DTC, the effect was greater for the dry weight, with 32.1% inhibition, whereas for the number of fronds, the effect was 17.5%, which is considered a non-toxic effect. DMSO 0.5% (*v*/*v*) in water also had a significant effect. Although we may consider that DMSO may have contributed to the inhibitory effect caused by the DTC, it is important to highlight that it was diluted in all the concentrations of DTC tested. In addition to the growth-inhibiting effect, some morphological differences in the fronds were noted. The inhibition effect at 150 mg L^−1^ was greater on the dry weight (24.4%) for the MSiNPs than on the number of fronds (18.6%), while for the MSiNP-DTC, the opposite was observed, with 22% and 35.2%, respectively. Although this change in toxicity is not remarkable, this once again may have been associated with the agglomeration/aggregation of the nanoparticles in the Steinberg medium, at least for the loaded NPs. As can be seen in Table 1, there was a considerable reduction in the ZP values compared to water, followed by an increase in the Z-Ave values mainly for the MSiNP-DTC. The aggregation may have caused the superficial attachment of the particles to the walls of the plant cells with strong adhesion, preventing the *L. minor* from having enough food and nutrition [63].

## 6. Conclusions

In conclusion, the DTC compound was successfully synthesized and loaded in MSiNPs by means of a simple and efficient synthesis. The characterization of the obtained MSiNPs shows that the synthesized nanoparticles had homogeneous sizes suitable for drug delivery systems. Concerning the loading efficiency of the MSiNPs, the DTC was successfully adsorbed to the MSiNPs, and these results suggest that MSiNPs have potential for the delivery of DTC in particular, and possibly for wider use in drug delivery applications, improving the stability and overcoming the low water solubility of Schiff’s dithiocarbazate bases. Paired with the development of MSiNP-DTC, it is also essential to clearly understand the potential risks of these systems to the environment. Based on the results obtained, it can be considered that the DTC, MSiNPs, and MSiNP-DTC did not show remarkable toxicity to aquatic organisms at the range of concentrations tested. Future work should include the testing of DTC, MSiNPs, and MSiNPs with other species, including terrestrial ones, and with human cell lines to better assure their safety. Afterwards, the activity/efficacy of these MSiNPs loaded with DTC can be tested for different applications already suggested for Schiff bases.

## Figures and Tables

**Table 1:** Average hydrodynamic size ± standard deviation (Z-Ave ± SD), polydispersity index (PDI ± SD), and average zeta potential (ZP ± SD) recorded for MSiNPs and MSiNP-DTC dispersed at 200 mg L^−1^ in water and in different assays’ culture media.

SAMPLES	MEDIA	Z-AVE ± SD/NM	PDI ± SD	ZP ± SD/MV
MSiNP	Water	168 ± 4	0.29 ± 0.02	−11.7 ± 0.4
	MBL	186 ± 17	0.39 ± 0.03	−30.1 ± 0.3
	Steinberg	189 ± 6	0.45 ± 0.03	−10.3 ± 0.4
MSiNP-DTC	Water	176 ± 1	0.38 ± 0.04	−21.9 ± 0.3
	MBL	200 ± 9	0.41 ± 0.04	−29.1 ± 0.4
	Steinberg	282 ± 31	0.47 ± 0.05	−11.0 ± 0.4

**Table 2:** *A. fischeri* toxicity results obtained from bioluminescence test (in mg L^−1^) after exposure to DTC, MSiNPs, and MSiNP-DTC at 5, 15, and 30 min of exposure (95% confidence interval, inside parentheses). Data are highest effect (%) expressed as bioluminescence inhibition percentages and EC_50_/EC_20_ values.

Samples	EC_50_/(mg L^−1^)			EC_20_/(mg L^−1^)		
	5 min	15 min	30 min	5 min	15 min	30 min
DTC	NC (h.e. 6.2%)	NC (h.e. 6.5%)	NC (h.e. 2.6%)	NC (h.e. 6.2%)	NC (h.e. 6.5%)	NC (h.e. 2.6%)
MSiNPs	NC (h.e. 17.4%)	NT	NC (h.e. 8.5%)	NC (h.e. 17.4%)	NC (h.e. 13.4%)	NC (h.e. 8.5%)
MSiNP-DTC	NC (h.e. 11.5%)	NC (h.e. 8.7%)	NC (h.e. 13.9%)	NC (h.e. 6.2%)	NC (h.e. 8.7%)	NC (h.e. 46.4%)

Note. DTC: 3-methyl-5-phenyl-pyrazoline-1-(*S*-benzyldithiocarbazate); MSiNPs: mesoporous silica nanoparticles; MSiNP-DTC: DTC-loaded silica nanoparticles; EC_50_/EC_20_: effect concentrations causing 50 or 20% of bioluminescence inhibition, respectively; NC: could not be calculated; NT: not toxic; h.e.: highest effect. The concentrations of MSiNP-DTC are based on MSiNPs. The amount of DTC in 150 mg L^−1^ of MSiNPs was 7.18 mg L^−1^.

**Table 3:** Effective concentrations (EC_50_/EC_20_), LOEC, and NOEC in mg L^−1^ of DTC, MSiNPs, and MSiNP-DTC for the microalgae *R. subcapitata* after 72 h of exposure and for *L. minor* after 7 days of exposure (CI means the lower and upper 95% confidence interval, inside parentheses).

BIOTA	ENDPOINT	SAMPLES	EC50/MG L^−1^	EC20/MG L^−1^	LOEC/MG L^−1^	NOEC/MG L^−1^
*R.* *SUBCAPITATA*	Growth rate	DTC	>150.0	119.9 (106.4–133.4)	8.8	19.8
		MSiNPs		>150.0	19.8	8.8
		MSiNP-DTC		>150.0	66.7	44.4
*L. MINOR*	Growth rate (frond number)	DTC	>150.0	>150.0	66.7	44.4
		MSiNPs		>150.0	66.7	44.4
		MSiNP-DTC		81.3 (65.7–96.9)	44.4	29.6
*L. MINOR*	Growth rate (dry weight)	DTC	>150.0	114.6 (95.0–134.2)	66.7	44.4
		MSiNPs		137.8 (119.0–156.5)	29.6	19.8
		MSiNP-DTC		147.9 (132.6–163.1)	100	66.7

Note. DTC: 3-methyl-5-phenyl-pyrazoline-1-(*S*-benzyldithiocarbazate); MSiNPs: mesoporous silica nanoparticles; MSiNP-DTC: DTC-loaded silica nanoparticles. EC_50_/EC_20_ values: effect concentrations causing 50 or 20% of growth inhibition, respectively; LOEC: low observed effect concentration; NOEC: no observed effect concentration. The concentrations of MSiNP-DTC are based on MSiNPs. The amount of DTC in 150 mg L^−1^ of MSiNPs is 7.18 mg L^−1^.

## Data Availability

Not applicable.

## References

[1] Ghosh P., Dey S.K., Ara M.H., Karim K., Islam A.N. (2019). A review on synthesis and versatile applications of some selected Schiff bases with their transition metal complexes. Egypt. J. Chem..

[2] Zangrando E., Begum M., Sheikh M., Miyatake R., Hossain M., Alam M., Hasnat M., Halim M., Ahmed S., Rahman M. (2017). Synthesis, characterization, density functional study and antimicrobial evaluation of a series of bischelated complexes with a dithiocarbazate Schiff base ligand. Arab. J. Chem..

[3] Costa A.R., França R.N., Silva-Jardim I., Silva R.J.S., Lima-Santos J., Salay L.C., Santos R.L.S.R. (2021). Self-assembling micellar system based on Pluronic and pyrazole-dithiocarbazate-conjugate stimulates production of nitric oxide from macrophages. Colloid Interface Sci. Commun..

[4] de Menezes T.I., Costa R.D.O., Sanches R.N.F., Silva D.D.O., Santos R.L.S.R. (2019). Preparation and characterization of dithiocarbazate Schiff base–loaded poly(lactic acid) nanoparticles and analytical validation for drug quantification. Colloid Polym. Sci..

[5] Costa A.R., de Menezes T.I., Nascimento R.R., dos Anjos P.N.M., Viana R.B., Fernandes A.G.D.A., Santos R.L.S.R. (2019). Ruthenium(II) dimethylsulfoxide complex with pyrazole/dithiocarbazate ligand. J. Therm. Anal. Calorim..

[6] Khan M.F., Alam M.M., Verma G., Akhtar W., Akhter M., Shaquiquzzaman M. (2016). The therapeutic voyage of pyrazole and its analogs: A review. Eur. J. Med. Chem..

[7] Freitas L.B.D.O., Bravo I.J.G., Macedo W.A.D.A., de Sousa E.M.B. (2015). Mesoporous silica materials functionalized with folic acid: Preparation, characterization and release profile study with methotrexate. J. Sol-Gel Sci. Technol..

[8] Safari J., Zarnegar Z. (2014). Advanced drug delivery systems: Nanotechnology of health design A review. J. Saudi Chem. Soc..

[9] Mamaeva V., Sahlgren C., Lindén M. (2013). Mesoporous silica nanoparticles in medicine—Recent advances. Adv. Drug Deliv. Rev..

[10] Tella J.O., Adekoya J.A., Ajanaku K.O. (2022). Mesoporous silica nanocarriers as drug delivery systems for anti-tubercular agents: A review. R. Soc. Open Sci..

[11] Moodley T., Singh M. (2021). Current Stimuli-Responsive Mesoporous Silica Nanoparticles for Cancer Therapy. Pharmaceutics.

[12] Pérez-Garnes M., Gutiérrez-Salmerón M., Morales V., Chocarro-Calvo A., Sanz R., García-Jiménez C., García-Muñoz R.A. (2020). Engineering hollow mesoporous silica nanoparticles to increase cytotoxicity. Mater. Sci. Eng. C.

[13] Chircov C., Spoială A., Păun C., Crăciun L., Ficai D., Ficai A., Andronescu E., Turculeƫ C. (2020). Mesoporous Silica Platforms with Potential Applications in Release and Adsorption of Active Agents. Molecules.

[14] Stober W., Fink A., Bohn E. (1968). Controlled growth of monodisperse silica spheres in the micron size range. J. Colloid Interface Sci..

[15] Hassan S., Prakash G., Ozturk A., Saghazadeh S., Sohail F., Seo J., Dockmeci M., Zhang Y.S., Arabia S. (2018). Evolution and clinical translation of drug delivery nanomaterials. HHS Public Access.

[16] Andreani T., Nogueira V., Gavina A., Fernandes S., Rodrigues J.L., Pinto V.V., Ferreira M.J., Silva A.M., Pereira C.M., Pereira R. (2020). Ecotoxicity to Freshwater Organisms and Cytotoxicity of Nanomaterials: Are We Generating Sufficient Data for Their Risk Assessment?. Nanomaterials.

[17] Fekete-Kertész I., Maros G., Gruiz K., Molnár M. (2016). The Effect of TiO 2 Nanoparticles on the Aquatic Ecosystem: A Comparative Ecotoxicity Study with Test Organisms of Different Trophic Levels. Period. Polytech. Chem. Eng..

[18] Ríos F., Fernández-Arteaga A., Fernández-Serrano M., Jurado E., Lechuga M. (2018). Silica micro- and nanoparticles reduce the toxicity of surfactant solutions. J. Hazard. Mater..

[19] Book F., Ekvall M.T., Persson M., Lönnerud S., Lammel T., Sturve J., Backhaus T. (2019). Ecotoxicity screening of seven different types of commercial silica nanoparticles using cellular and organismic assays: Importance of surface and size. Nanoimpact.

[20] Liu P., Zhao Y., Wang S., Xing H., Dong W.-F. (2021). Effect of combined exposure to silica nanoparticles and cadmium chloride on female zebrafish ovaries. Environ. Toxicol. Pharmacol..

[21] Gürol M.A., Arman S., Yön N.D. (2020). Effects of mancozeb on the testicular histology of the zebrafish (Danio rerio). Ann. Limnol. Int. J. Limnol..

[22] Paganotto Leandro L., Siqueira de Mello R., da Costa-Silva D.G., Medina Nunes M.E., Rubin Lopes A., Kemmerich Martins I., Posser T., Franco J.L. (2021). Behavioral Changes Occur Earlier than Redox Alterations in Developing Zebrafish Exposed to Mancozeb. Environ. Pollut..

[23] Tilton F., La Du J.K., Vue M., Alzarban N., Tanguay R.L. (2006). Dithiocarbamates have a common toxic effect on zebrafish body axis formation. Toxicol. Appl. Pharmacol..

[24] Chen X., Fang M., Chernick M., Wang F., Yang J., Yu Y., Zheng N., Teraoka H., Nanba S., Hiraga T. (2018). The case for thyroid disruption in early life stage exposures to thiram in zebrafish (Danio rerio). Gen. Comp. Endocrinol..

[25] De Sousa G.F., Gatto C.C., Resck I.S., Deflon V.M. (2010). Synthesis, Spectroscopic Studies and X-ray Crystal Structures of New Pyrazoline and Pyrazole Derivatives. J. Chem. Crystallogr..

[26] Paula A.J., Martinez D.S.T., Júnior R.T.A., Filho A.G.S., Alves O.L. (2012). Suppression of the hemolytic effect of mesoporous silica nanoparticles after protein corona interaction: Independence of the surface microchemical environment. J. Braz. Chem. Soc..

[27] Brunauer S., Emmett P.H., Teller E. (1938). Adsorption of Gases in Multimolecular Layers. J. Am. Chem. Soc..

[28] Barrett E.P., Joyner L.G., Halenda P.P. (1951). The Determination of Pore Volume and Area Distributions in Porous Substances. I. Computations from Nitrogen Isotherms. J. Am. Chem. Soc..

[29] Andreani T., Fernandes P.M.V., Nogueira V., Pinto V.V., Ferreira M.J., Rasteiro M.G., Pereira R., Pereira C.M. (2020). The critical role of the dispersant agents in the preparation and ecotoxicity of nanomaterial suspensions. Environ. Sci. Pollut. Res..

[30] Andreani T., Nogueira V., Pinto V.V., Ferreira M.J., Rasteiro M.G., Silva A.M., Pereira R., Pereira C.M. (2017). Influence of the stabilizers on the toxicity of metallic nanomaterials in aquatic organisms and human cell lines. Sci. Total. Environ..

[31] Microbics Corporation (1998). Azur Environmental Microtox^®^ Omni Manual.

[32] OECD 201 (2006). Organisation for Economic Co-operation and Development (OECD)Guidelines for testing of chemicals. Test No. 201: Freshwater Algae and Cyanobacteria, Growth Inhibition Test.

[33] OECD 221 (2006). Organisation for Economic Co-operation and Development Guidelines for testing of chemicals. Test No. 221: Lemna sp. Growth Inhibition Test.

[34] Vale G., Mehennaoui K., Cambier S., Libralato G., Jomini S., Domingos R.F. (2016). Manufactured nanoparticles in the aquatic environment-biochemical responses on freshwater organisms: A critical overview. Aquat. Toxicol..

[35] Lekamge S., Miranda A.F., Abraham A., Ball A.S., Shukla R., Nugegoda D. (2020). The toxicity of coated silver nanoparticles to the alga Raphidocelis subcapitata. SN Appl. Sci..

[36] Gorbachuk V.V., Yakimova L.S., Mostovaya O.A., Bizyaev D.A., Bukharaev A.A., Antipin I.S., Konovalov A.I., Zharov I., Stoikov I.I. (2011). Silica Nanoparticles with Proton Donor and Proton Acceptor Groups: Synthesis and Aggregation. Silicon.

[37] Sherif O.E., Issa Y.M., Abbas S.M. (2000). Thermodynamic Parameters of Some Schiff Bases Derived From 5,7-dihydroxy-6-formyl-2-methylbenzopyran-4-one. J. Therm. Anal. Calorim..

[38] Awad E., El-Fiqi A., Austin D., Lyndon A. (2020). Possible effect of lesser galangal (*Alpinia officinarum*) extracts encapsulated into mesoporous silica nanoparticles on the immune status of rainbow trout (*Oncorhynchus n*). Aquac. Res..

[39] Arriagada F., Günther G., Nos J., Nonell S., Olea-Azar C., Morales J. (2019). Antioxidant Nanomaterial Based on Core–Shell Silica Nanospheres with Surface-Bound Caffeic Acid: A Promising Vehicle for Oxidation-Sensitive Drugs. Nanomaterials.

[40] Nhavene E.P.F., da Silva W.M., Junior R.R.T., Gastelois P.L., Venâncio T., Nascimento R., Batista R.J.C., Machado C.R., Macedo W.A.D.A., de Sousa E.M.B. (2018). Chitosan grafted into mesoporous silica nanoparticles as benznidazol carrier for Chagas diseases treatment. Microporous Mesoporous Mater..

[41] Enache D.F., Vasile E., Simonescu C.M., Culita D., Oprea O., Pandele A.M., Razvan A., Dumitru F., Nechifor G. (2018). Schiff base-functionalized mesoporous silicas (MCM-41, HMS) as Pb(ii) adsorbents. RSC Adv..

[42] Nikoorazm M., Ghorbani-Choghamarani A., Noori N. (2015). Oxo-vanadium(IV) Schiff base complex supported on modified MCM-41: A reusable and efficient catalyst for the oxidation of sulfides and oxidative S-S coupling of thiols. Appl. Organomet. Chem..

[43] Ghaferi M., Zahra S.W., Shahmabadi H.E., Alavi S.E. (2021). Enhancing the Efficacy of Albendazole for Liver Cancer Treatment Using Mesoporous Silica Nanoparticles: An in Vitro Study. EXCLI J..

[44] Hegazy M., Zhou P., Wu G., Wang L., Rahoui N., Taloub N., Huang X., Huang Y. (2017). Construction of polymer coated core–shell magnetic mesoporous silica nanoparticles with triple responsive drug delivery. Polym. Chem..

[45] Sachar H.S., Sivasankar V.S., Das S. (2019). Electrostatics and Interactions of an Ionizable Silica Nanoparticle Approaching a Plasma Membrane. Langmuir.

[46] Wahab M., Imae I., Kawakami Y., Kim I., Ha C.-S. (2006). Functionalized periodic mesoporous organosilica fibers with longitudinal pore architectures under basic conditions. Microporous Mesoporous Mater..

[47] Talavera-Pech W.A., Esparza-Ruiz A., Quintana-Owen P., Vilchis-Nestor A.R., Carrera-Figueiras C., Ávila-Ortega A. (2016). Effects of different amounts of APTES on physicochemical and structural properties of amino-functionalized MCM-41-MSNs. J. Sol-Gel Sci. Technol..

[48] Popova M.D., Szegedi Á., Kolev I.N., Mihály J., Tzankov B.S., Momekov G.T., Lambov N.G., Yoncheva K.P. (2012). Carboxylic modified spherical mesoporous silicas as drug delivery carriers. Int. J. Pharm..

[49] Wahab M.A., Imae I., Kawakami Y., Ha C.-S. (2005). Periodic Mesoporous Organosilica Materials Incorporating Various Organic Functional Groups: Synthesis, Structural Characterization, and Morphology. Chem. Mater..

[50] Speed D., Westerhoff P., Sierra-Alvarez R., Draper R., Pantano P., Aravamudhan S., Chen K.L., Hristovski K., Herckes P., Bi X. (2015). Physical, chemical, and in vitro toxicological characterization of nanoparticles in chemical mechanical planarization suspensions used in the semiconductor industry: Towards environmental health and safety assessments. Environ. Sci. Nano.

[51] Casado M.P., Macken A., Byrne H.J. (2013). Ecotoxicological assessment of silica and polystyrene nanoparticles assessed by a multitrophic test battery. Environ. Int..

[52] Gao Q., Wang J., Ren L., Cheng Y., Lin Z., Li X.-G., Sun H. (2021). Investigations on the influence of energy source on time-dependent hormesis: A case study of sulfadoxine to Aliivibrio fischeri in different cultivation systems. Sci. Total. Environ..

[53] Budragchaa T., Westermann B., Wessjohann L.A. (2019). Multicomponent synthesis of α-acylamino and α-acyloxy amide derivatives of desmycosin and their activity against gram-negative bacteria. Bioorganic Med. Chem..

[54] Nguyen M.K., Moon J.-Y., Lee Y.-C. (2020). Microalgal ecotoxicity of nanoparticles: An updated review. Ecotoxicol. Environ. Saf..

[55] Lomba L., Lapeña D., Ros N., Aso E., Cannavò M., Errazquin D., Giner B. (2020). Ecotoxicological study of six drugs in Aliivibrio fischeri, Daphnia magna and Raphidocelis subcapitata. Environ. Sci. Pollut. Res..

[56] Wang F., Guan W., Xu L., Ding Z., Ma H., Ma A., Terry N. (2019). Effects of Nanoparticles on Algae: Adsorption, Distribution, Ecotoxicity and Fate. Appl. Sci..

[57] de Beukelaar M.F., Zeinstra G.G., Mes J.J., Fischer A.R. (2019). Duckweed as human food. The influence of meal context and information on duckweed acceptability of Dutch consumers. Food Qual. Preference.

[58] Pagliuso D., Grandis A., Fortirer J.S., Camargo P., Floh E.I.S., Buckeridge M.S. (2022). Duckweeds as Promising Food Feedstocks Globally. Agronomy.

[59] Baek G., Saeed M., Choi H.-K. (2021). Duckweeds: Their utilization, metabolites and cultivation. Appl. Biol. Chem..

[60] Chen G., Zhao K., Li W., Yan B., Yu Y., Li J., Zhang Y., Xia S., Cheng Z., Lin F. (2022). A review on bioenergy production from duckweed. Biomass Bioenergy.

[61] Modlitbová P., Hlaváček A., Švestková T., Pořízka P., Šimoníková L., Novotný K., Kaiser J. (2019). The effects of photon-upconversion nanoparticles on the growth of radish and duckweed: Bioaccumulation, imaging, and spectroscopic studies. Chemosphere.

[62] Park J., Yoo E.-J., Shin K., Depuydt S., Li W., Appenroth K.-J., Lillicrap A.D., Xie L., Lee H., Kim G. (2021). Interlaboratory Validation of Toxicity Testing Using the Duckweed *Lemna minor* Root-Regrowth Test. Biology.

[63] Goswami L., Kim K.-H., Deep A., Das P., Bhattacharya S.S., Kumar S., Adelodun A.A. (2017). Engineered nano particles: Nature, behavior, and effect on the environment. J. Environ. Manag..

